# Comparison of Bloodmeal Digestion and the Peritrophic Matrix in Four Sand Fly Species Differing in Susceptibility to *Leishmania donovani*

**DOI:** 10.1371/journal.pone.0128203

**Published:** 2015-06-01

**Authors:** Katerina Pruzinova, Jovana Sadlova, Veronika Seblova, Miroslav Homola, Jan Votypka, Petr Volf

**Affiliations:** Department of Parasitology, Faculty of Science Charles University, Prague, Czech Republic; Universidade Federal do Rio de Janeiro, BRAZIL

## Abstract

The early stage of *Leishmania* development in sand flies is closely connected with bloodmeal digestion. Here we compared various parameters of bloodmeal digestion in sand flies that are either susceptible (*Phlebotomus argentipes* and *P*. *orientalis*) or refractory (*P*. *papatasi* and *Sergentomyia schwetzi*) to *Leishmania donovani*, to study the effects on vector competence. The volume of the bloodmeal ingested, time of defecation of bloodmeal remnants, timing of formation and degradation of the peritrophic matrix (PM) and dynamics of proteolytic activities were compared in four sand fly species. Both proven vectors of *L*. *donovani* showed lower trypsin activity and slower PM formation than refractory species. Interestingly, the two natural *L*. *donovani* vectors strikingly differed from each other in secretion of the PM and midgut proteases, with *P*. *argentipes* possessing fast bloodmeal digestion with a very high peak of chymotrypsin activity and rapid degradation of the PM. Experimental infections of *P*. *argentipes* did not reveal any differences in vector competence in comparison with previously studied *P*. *orientalis*; even the very low initial dose (2×103 promastigotes/ml) led to fully developed late-stage infections with colonization of the stomodeal valve in about 40% of females. We hypothesise that the period between the breakdown of the PM and defecation of the bloodmeal remnants, i.e. the time frame when *Leishmania* attach to the midgut in order to prevent defecation, could be one of crucial parameters responsible for the establishment of *Leishmania* in the sand fly midgut. In both natural *L*. *donovani* vectors this period was significantly longer than in *S*. *schwetzi*. Both vectors are equally susceptible to *L*. *donovani*; as average bloodmeal volumes taken by females of *P*. *argentipes* and *P*. *orientalis* were 0.63 μl and 0.59 μl, respectively, an infective dose corresponding to 1–2 parasites was enough to initiate mature infections.

## Introduction

The parasitic protozoan *Leishmania donovani* (Kinetoplastida: Trypanosomatidae) causes visceral leishmaniasis, also called kala-azar, which is a serious health problem in the Indian subcontinent and East Africa. This *Leishmania* species is transmitted by sand flies of the genus *Phlebotomus*, the most important vectors being *Phlebotomus orientalis* and *P*. *argentipes* (reviewed by [1,2]).

In sand fly vectors, the development of *Leishmania* parasites is confined to the digestive tract. The early phase of infection is closely associated with bloodmeal digestion as parasites multiply and morphologically transform in the lumen of midgut, within the bloodmeal surrounded by the peritrophic matrix (PM). Parasites that successfully develop and survive this early stage must then attach to the midgut epithelium and establish late-stage infections (for review see [3–6]).

One of the first obstacles parasites must surpass in the sand fly midgut is the effect of midgut digestive enzymes, particularly trypsin- and chymotrypsin-like proteases. Production of these serine proteases by midgut epithelial cells starts several hours post bloodmeal (PBM) and their activities peak at 18–48 hours PBM, depending on the sand fly species [7,8].

It has been repeatedly published that proteolytic activities of midgut proteases influence *Leishmania* development and the vector competence of sand flies [9–11]. Adler [12] was the first who suggested that products of blood serum digestion destroy *Leishmania* parasites in the midguts of ‘noncompatible’ sand fly species. According to Schlein and Romano [13] and Borovsky and Schlein [9], specific components of the trypsin-like activity prevents the survival of *L*. *donovani* in the ‘noncompatible’ vector *P*. *papatasi* while the ability to modulate this factor enables *L*. *major* to survive in ‘compatible’ sand fly species. It has been shown *in vitro* that midgut lysates of blood-fed *P*. *papatasi* (a natural vector of *L*. *major*) kills *Leishmania* transforming from the amastigote to promastigote stage, and this effect was attributed to midgut proteases [11]. Several studies have also described that in natural vector-parasite combinations, *Leishmania* modulate the levels of insect digestive enzymes at both the protein and transcriptional levels [8,13,14]. In the New World sand fly *Lutrzomyia longipalpis*, Santos *et al*. [15] recently described that *L*. *infantum* modulates trypsin activity, supposingly by decreasing the pH in the midgut.

A second potential barrier for *Leishmania* infection is the peritrophic matrix (PM). The peritrophic matrix is an acellular semipermeable envelope that separates ingested blood from the midgut epithelium and compartmentalizes digestion between endo- and ectoperitrophic spaces. This structure is composed of chitin, proteins and glycoproteins, and plays a key role in protection of the midgut epithelium from pathogens and chemical and mechanical damage (reviewed by [16]). In mosquitoes and other hematophagous insects, the PM performs a central role in heme detoxification [17] and its disruption affects fecundity of sand fly females [18].

The role of the PM in *Leishmania*—sand fly interactions seems to be twofold: the PM has been suggested to protect parasites from proteolytic damage within amastigote-to-promastigote transition [11]; on the other hand, it creates a physical barrier that prevents the early escape of *Leishmania* parasites into the ectoperitrophic space, which may result in their defecation with bloodmeal remnants (reviewed by [3,5]). Recently, Sadlova and Volf [19] demonstrated that *Leishmania major* in its natural vector *P*. *duboscqi* stays in the intraperitrophic space until the PM is broken by sand fly-derived chitinases and only then colonize the ectoperitrophic space of the midgut. Finally, after successful escape into ectoperitrophic space, promastigotes must overcome a critical period of defecation by inserting flagella between microvilli of the midgut epithelium (reviewed by [4,20–22]). As the process of formation and degradation of the PM in blood sucking insects is highly species-specific (reviewed by [16]), the formation and persistence of the PM seems to be one of main potential factors involved in the vector competence of sand flies.

The main aim of the work was a thorough understanding of the factors in the sand fly midgut which *Leishmania* parasites meet during the early stage of infection. Detailed study of midgut proteolytic activity and development of the peritrophic matrix in several vector species differing in vector competence was expected to clarify which factors differ interspecifically and can cause failure of the parasite development. Therefore, we selected two sand fly species which fully support development of *L*. *donovani* (*P*. *orientalis* and *P*. *argentipes*, reviewed by [1,2]) and two species which are refractory to this parasite (*S*. *schwetzi* and *P*. *papatasi* [9,10,23,24]) and studied the bloodmeal volume imbibed by females, the time of defecation of bloodmeal remnants, timing of the formation and degradation of the PM and dynamics of midgut protease activities. In addition, we tested dose-dependent differences in *L*. *donovani* development in its natural vectors and the number of parasites required for a successful infection.

One would expect that two *L*. *donovani* vectors do not differ significantly each other in main aspects of bloodmeal digestion and development of the PM. However, we found the opposite; there is striking difference in all parameters studied and bloodmeal digestion of *P*. *orientalis* is, in some aspects, more similar to *P*. *papatasi* than to *P*. *argentipes*.

## Methods

### Sand fly maintenance

Colonies of *Phlebotomus argentipes*, *P*. *orientalis*, *P*. *papatasi* and *Sergentomyia schwetzi* were maintained under standard conditions as previously described [25]. Three to seven day old females were used in all experiments and were maintained at 26°C on 50% sucrose.

### Haemoglobin assay for measuring the bloodmeal volume

During bloodfeeding, sand fly females expel a significant volume of water by prediuresis [26]; consequently, the classical method of weighing bloodfed females leads to an underestimation of the bloodmeal volume. Therefore, the colorimetric method by Briegel *et al*. [27], developed for measuring the haemoglobin concentration in bloodfed mosquitoes, was adopted to study the bloodmeal volume ingested by sand fly females. All studied sand fly species were fed on anesthetized BALB/c mice (Experiment A) or on heat-inactivated rabbit blood offered by membrane feeding (using a chick-skin membrane; Experiment B). Individual guts of fully bloodfed females were dissected 1h after bloodfeeding, transferred to tubes containing 200 μl 0.15 mM NaCl and homogenized. Twenty dissected guts of each sand fly species were used in two independent repetitions of each experiment. Volumes of 50 μl of the gut homogenates or diluted blood (5 μl fresh mouse blood or heat-inactivated rabbit blood in 1000 μl 0.15 mM NaCl) were mixed with 200 μl of Drabkin’s reagent (Sigma) in the dark for 30 min. Absorbance was measured in 96-well plate in doublets at 540 nm. Human haemoglobin (Sigma) at concentrations from 3.1 to 100μg/well was used as a standard. Data from two independent repetitions were pooled and statistical evaluations were performed by the ANOVA and Post-Hoc Tukey HSD test using STATISTICA 12 software.

### Sand fly defecation

Females of *P*. *argentipes*, *P*. *orientalis*, *P*. *papatasi* and *S*. *schwetzi* were fed on anesthetized BALB/c mice (Experiment A) or through a chick-skin membrane on heat-inactivated rabbit blood (Experiment B), and a previously described method [28] was used to compare defecation times of the sand fly females. Briefly, fully bloodfed females were individually placed in small glass vials and checked twice a day under a binocular microscope for defecation.

### Timing of the formation and degradation of the PM

Bloodfed females of *P*. *argentipes*, *P*. *orientalis*, *P*. *papatasi* and *S*. *schwetzi* were dissected at ten intervals after feeding on anesthetized BALB/c mice, starting immediately PBM and at each of the following times: 1, 3, 6, 12, 24, 48, 72, 96, and 120h PBM, and checked for the formation, breakdown and degradation of the PM. Dissections were carried out in isotonic saline solution with brief washing of the gut in distilled water in order to better separate the PM [29]. For each studied sand fly species and time interval, at least 20 females were analyzed. Slides were observed under an Olympus BX51 microscope with Nomarski contrast and photographed with an Olympus D70 camera and software. The PM was classified as i) intact, when no traces of blood escape were observed, ii) broken, when blood started to escape to the ectoperitrophic space and iii) degraded, when it was disintegrated and defecated.

### Fluorometric assay for quantification of proteolytic activities

Midgut trypsin and chymotrypsin activities were measured in *P*. *argentipes*, *P*. *orientalis*, *P*. *papatasi* and *S*. *schwetzi* after feeding on anesthetized BALB/c mice (Experiment A) or heat-inactivated rabbit blood (Experiment B) via a chick-skin membrane. Midguts of fully bloodfed females were dissected at 12, 24, 36, 48, 72, 80, and 96 hours PBM and transferred to 1.5 ml Eppendorf tubes. In total, 2240 midguts were dissected and used for quantification of trypsin and chymotrypsin activities; for practical reasons, the activities were measured in pools of 10 midguts in 100 μl of Tris-NaCl (0.1 M Tris, 150 mM NaCl, pH = 8.44). The samples were homogenized, and trypsin and chymotrypsin activities were measured in a black 96-well plate by a fluorometric assay with the substrates Boc-Leu-Gly-Arg-AMC and Suc-Ala-Ala-Pro-Phe-AMC (40 μM; Bachem), respectively. Aminomethylcoumarin (AMC) was excited at 355 nm and the fluorescence of released AMC was measured at 460 nm by a fluorometer (Tecane infinite M200). Each point in the graphs represents a measurement of four pools (two from each experiment). The proteolytic activities are presented as rfu/μg of protein of mouse or rabbit blood. The concentration of substrate was optimised in the series of previous experiments, 40 μM concentration was found optimal, providing stable results. Each experiment was repeated twice. Total proteolytic activities were calculated as the area under the time curve.

### *Leishmania* parasites

*Leishmania donovani* strain GR374 (MHOM/ET/2010/DM-1033) was maintained at 23°C on Medium 199 (Sigma) supplemented with 10% foetal calf serum (Gibco), 1% BME vitamins (Sigma), 2% human urine and amikin (250 μg/ml).

### Experimental infections of *P*. *argentipes*: the effect of initial infective dose

The effect of different initial infective doses on *L*. *donovani* development was previously studied by our group in *P*. *orientalis* [30]. Here we tested the effect of initial infective doses on infection rates and total parasite numbers in *P*. *argentipes*, another major vector of *L*. *donovani*. In addition to the parasite dose generally used for sand fly infections (5×10^5^), two lower infective doses were tested. Females of *P*. *argentipes* were fed for one hour through a chick-skin membrane on heat-inactivated rabbit blood containing 2×10^3^, 2×10^4^ or 5×10^5^ promastigotes per ml and fully bloodfed females were separated. On days 2 and 6 post-infection (PI) the females were dissected and the individual guts were checked microscopically for the presence and localization of *Leishmania* promastigotes. Parasite loads were graded according to Myskova *et al*. [31] as light (< 100 parasites/gut), moderate (100 − 1000 parasites/gut), or heavy (> 1000 parasites/gut). Data were evaluated statistically by means of the Chi-square test using STATISTICA 12 software.

### Experimental infections of *P*. *orientalis* and *P*. *argentipes*: comparison of susceptibility to *L*. *donovani*

Females of *P*. *argentipes* and *P*. *orientalis* of the same age were infected simultaneously using the same parasite culture at a dose of 2×10^3^ promastigotes/ml. Females were dissected on days 6 and 8 PI and checked microscopically. Special attention was paid to colonization of the stomodeal valve as the precondition for successful transmission to a mammalian host (for review see [5]). Parasite loads and statistical evaluations were graded according to Myskova *et al*. [31] and as described above.

### Real-time PCR for quantification of *Leishmania* in sand flies

On day 8 PI the numbers of *Leishmania* parasites in individual females were counted using Q-PCR as described previously [31,32]. Briefly, experimental females were stored at −20°C and total DNA extraction was performed with a High Pure PCR Template Preparation Kit (Roche) according to the manufacturer’s instructions. Kinetoplast DNA was chosen as the molecular target with previously described specific primers (forward: 5´-CTTTTCTGGTCCTCCGGGTAGG-3´; reverse: 5´-CCACCCGGCCCTATTTTACACCAA-3) [32]. Q-PCR was performed by the SYBR Green detection method (iQSYBER Green Supermix, Bio-Rad, Hercules, CA) in Bio-Rad iCycler & iQ Real-Time PCR systems. Statistical evaluation was performed by the Kruskal-Wallis test using STATISTICA 12 software.

### Ethical statement

Animals were maintained and handled in the animal facility of Charles University in Prague in accordance with institutional guidelines and Czech legislation (Act No. 246/1992 and 359/2012 coll. on Protection of Animals against Cruelty in present statutes at large), which complies with all relevant EU guidelines for experimental animals. All experiments were approved by the Committee on the Ethics of Laboratory Experiments of the Charles University in Prague and were performed under the Certificate of Competency (Registration Number: CZ 00177).

## Results

### Bloodmeal volume

**Fig 1:**
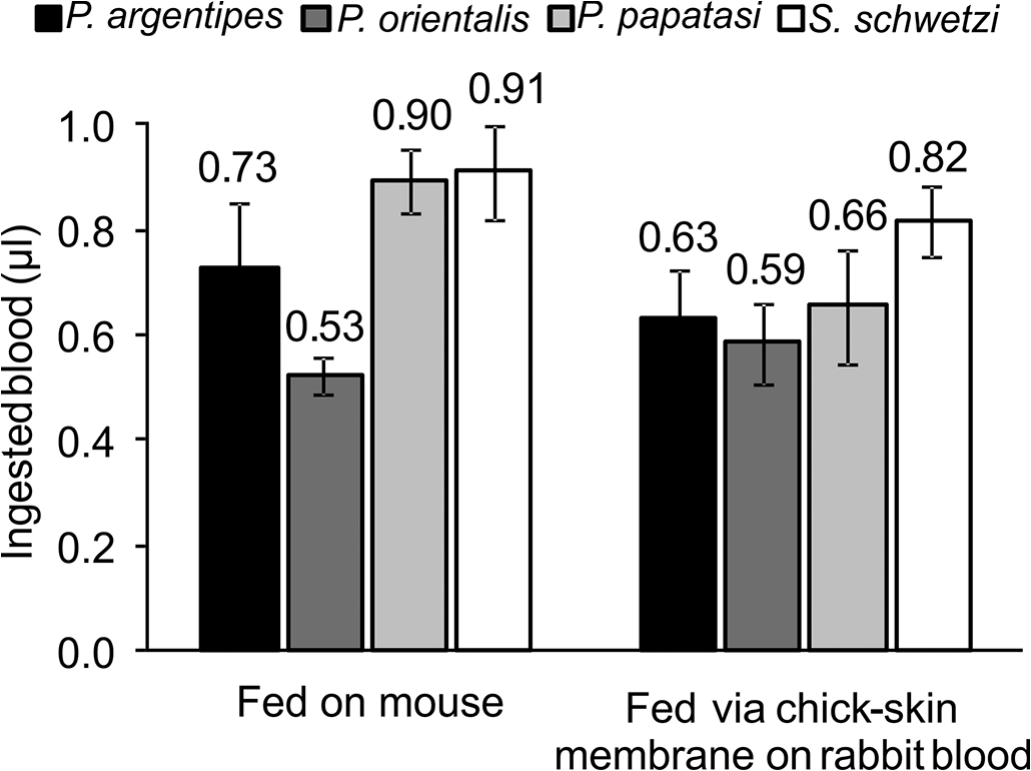
Bloodmeal volumes taken by *P*. *argentipes*, *P*. *orientalis*, *P*. *papatasi* and *S*. *schwetzi*. Females were fed on anesthetized BALB/c mice or through a chick-skin membrane on heat-inactivated rabbit blood and dissected 1 hour PBM. Bloodmeal volume was measured by a haemoglobin assay. Data from two independent repetitions were pooled; the bars indicate an average of forty dissected guts. The interspecific differences in bloodmeal volume taken by four sand fly species were highly significant (F_(3,27)_ = 11.62, P < 0.0001)

The interspecific differences in bloodmeal volume taken during feeding for the four sand fly species were highly significant (F_(3,27)_ = 11.62, P < 0.0001; Fig 1). Species refractory to *Leishmania donovani* (*P*. *papatasi* and *S*. *schwetzi*) took more blood than *L*. *donovani* vectors (*P*. *argentipes* and *P*. *orientalis*; F_(1,29)_ = 22.52, P < 0.0001). In three species the volume of ingested blood was significantly conditioned by the mode of feeding (F_(1,20)_ = 10.64, P < 0.005); on average, females of *P*. *argentipes*, *P*. *papatasi* and *S*. *schwetzi* ingested a larger volume of blood when feeding on anaesthetized mice (0.73 μl, 0.90 μl and 0.91 μl, respectively) than on rabbit blood via the chick-skin membrane (0.63 μl, 0.66 μl and 0.82 μl, respectively). In contrast, *P*. *orientalis* females took a similar volume of blood during membrane feeding and feeding on mice (0.59 μl vs. 0.53 μl; F_(1,6)_ = 1.58, P = 0.26). The Tukey’s Multiple Comparison Test table is provided as “Supporting information” (S1 Table). The bloodmeal volume does not necessarily correlate with body size of sand flies. For example *S*. *schwetzi* is smaller than *P*. *orientalis* but takes a bigger volume of bloodmeal.

### Sand fly defecation

**Fig 2:**
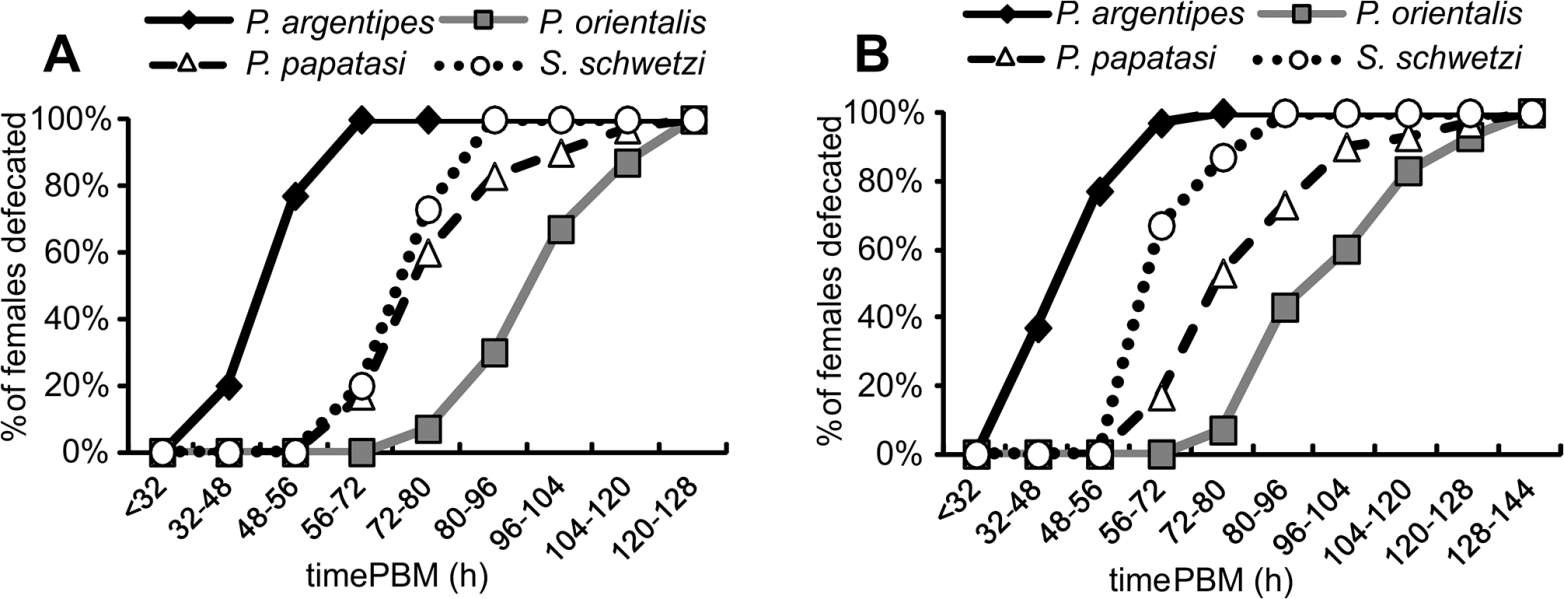
Defecation times of *P. argentipes*, *P. orientalis*, *P. papatasi* and *S*. *schwetzi*. Females were fed either on anesthetized BALB/c mice (**2A**) or on heat-inactivated rabbit blood via a chick-skin membrane (**2B**), individually placed in small glass vials and checked twice a day for defecation.

The fastest defecation was found in *P*. *argentipes*; all females defecated during an interval from 32 to 72 hours PBM. Conversely, the longest defecation time was found in *P*. *orientalis*; females started to defecate at the beginning of the fourth day PBM and 100% of females defecated by day five or six PBM. *Phlebotomus papatasi* and *S*. *schwetzi* females defecated from the end of the third till the fifth and sixth day PBM, respectively. In all sand fly species, the time courses of defecation did not differ between groups of females fed on mouse and those fed on rabbit blood through the chick-skin membrane (Fig 2).

### Timing of the formation and degradation of the PM

**Fig 3:**
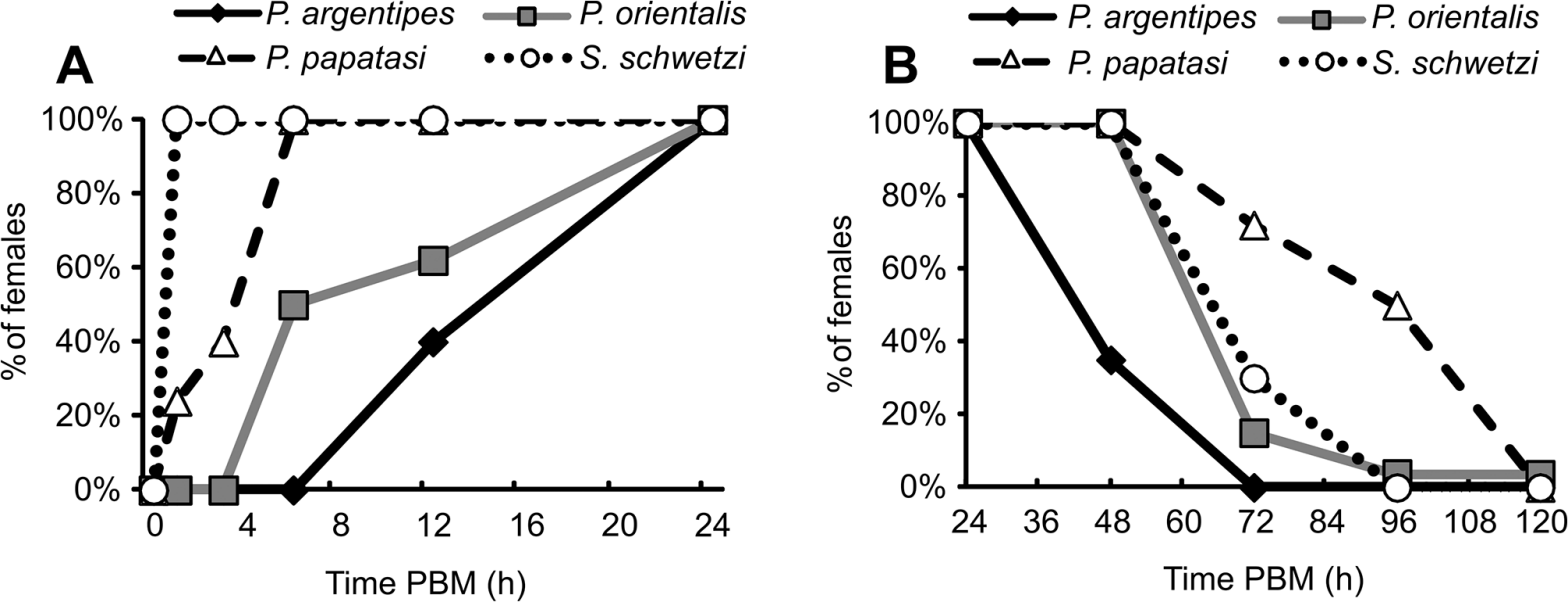
Timing of the peritrophic matrix (PM) formation and degradation in *P*. *argentipes*, *P*. *orientalis*, *P*. *papatasi* and *S*. *schwetzi*. Bloodfed females were dissected at 1, 3, 6, 12, 24, 48, 72, 96, and 120 hours PBM and checked for the formation (**3A**) and degradation (**3B**) of the PM.

**Fig 4:**
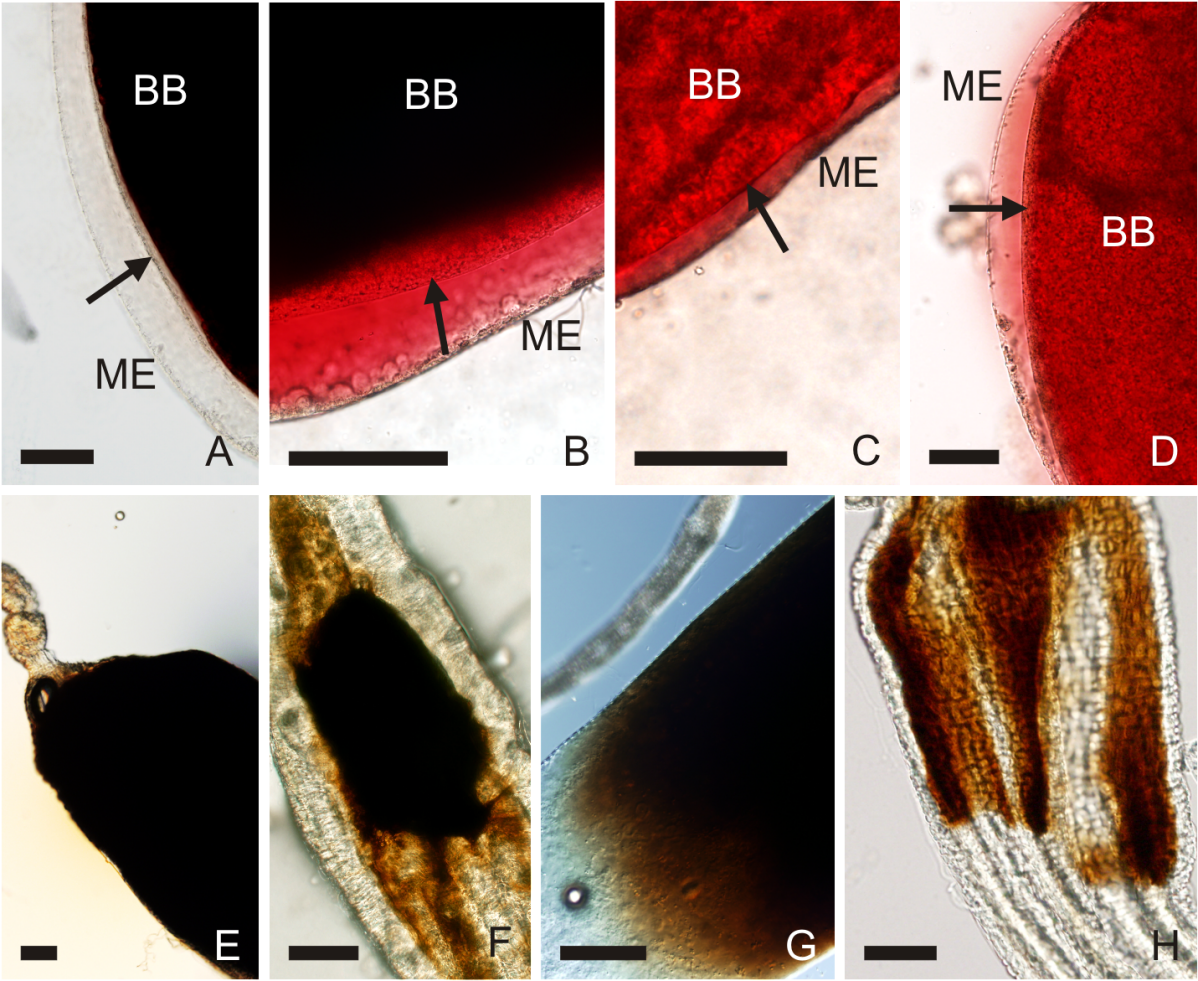
Formation (A-D) and degradation (E-H) of the peritrophic matrix (PM) in various sand fly species. The thin PM separating the blood bolus (BB) from the midgut epithelium (ME) is indicated by the arrow. It appears first by 1h PBM in *S*. *schwetzi* (4A) and *P*. *papatasi* (4B), 6 h PBM in *P*. *orientalis* (4C) and 12h PBM in *P*. *argentipes* (4D). At the end of digestion, the bloodmeal remnants are still enclosed by intact PM in *S*. *schwetzi* on day 3 PBM (4E). In contrast, in the other species studied bloodmeal remnants leak to abdominal midgut through broken PM, in *P*. *papatasi* typically by day 4 PBM (4F), in *P*. *orientalis* by day 3 PBM (4G) and in *P*. *argentipes* by day 2 PBM (4H). Scale bar = 50 μm.

Formation of the PM was extremely rapid in *S*. *schwetzi*, where all females developed the first thin PM within the first one hour PBM (Figs 3A and 4). Rapid formation was also detected in *P*. *papatasi*, where the first traces of the PM were present in 24% of females by 1h PBM (Fig 4) and a fully formed PM was present in 100% of females by 6h PBM. In contrast, formation of the PM was slower in *L*. *donovani* vectors; the first thin PM appeared by 6 and 12h PBM in *P*. *orientalis* and *P*. *argentipes*, respectively (Fig 4), and a fully formed PM was present in all females of both species by 24h PBM (Fig 3A).

Degradation of the PM was most rapid in *P*. *argentipes*, where only 35% of females possessed the PM by 48h PBM (Fig 4) and the PM was completely absent in all females by 72 hrs PBM (Fig 3B). In all three other species degradation of the PM started between the second and third day PBM. In *P*. *orientalis* and *S*. *schwetzi* the PM was present in 14% and 30% of females by day three PBM, respectively, and only in a few specimens in later time intervals (Fig 4). In *P*. *papatasi* the PM was present in 50% of females by day four PBM and it was not present in any females by day five PBM (Figs 3B and 4).

**Fig 5:**
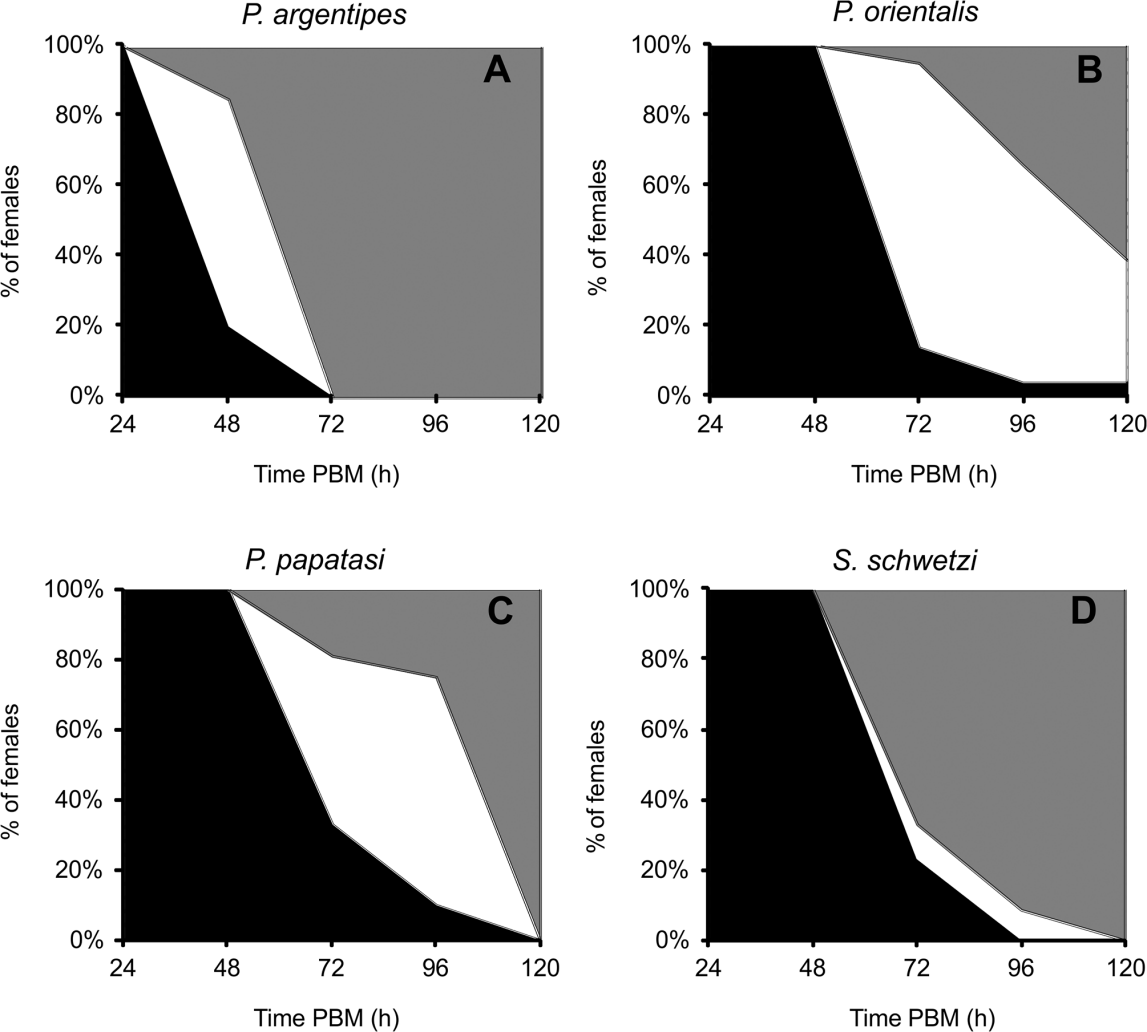
Timing of the peritrophic matrix (PM) breakdown and defecation of bloodmeal remnants in *P. argentipes*, *P. orientalis*, *P. papatasi* and *S*. *schwetzi*. Bloodfed females were dissected at 24, 48, 72, 96, and 120 hours PBM and checked for the localization of the bloodmeal and formation of the PM. **Black area:** females with bloodmeal enclosed inside the PM, **white area:** females with the PM broken or fully degraded where the bloodmeal is in contact with midgut epithelium, **grey area:** females after defecation of bloodmeal remnants. **5A:** *P*. *argentipes*, **5B:** *P*. *orientalis*, **5C:** *P*. *papatasi*, **5D:** *S*. *schwetzi*.

The period between the breakdown of the PM and defecation of bloodmeal remnants, i.e., the period when *Leishmania* promastigotes attach to the midgut epithelium in order to prevent defecation, strikingly differed among the four sand fly species (Fig 5). This time lasted on average 21h, 48h and 38h in *P*. *argentipes*, *P*. *orientalis* and *P*. *papatasi*, respectively, while in *S*. *schwetzi* it was significantly shorter (3h on average).

### Proteolytic activities in sand fly midguts

**Fig 6:**
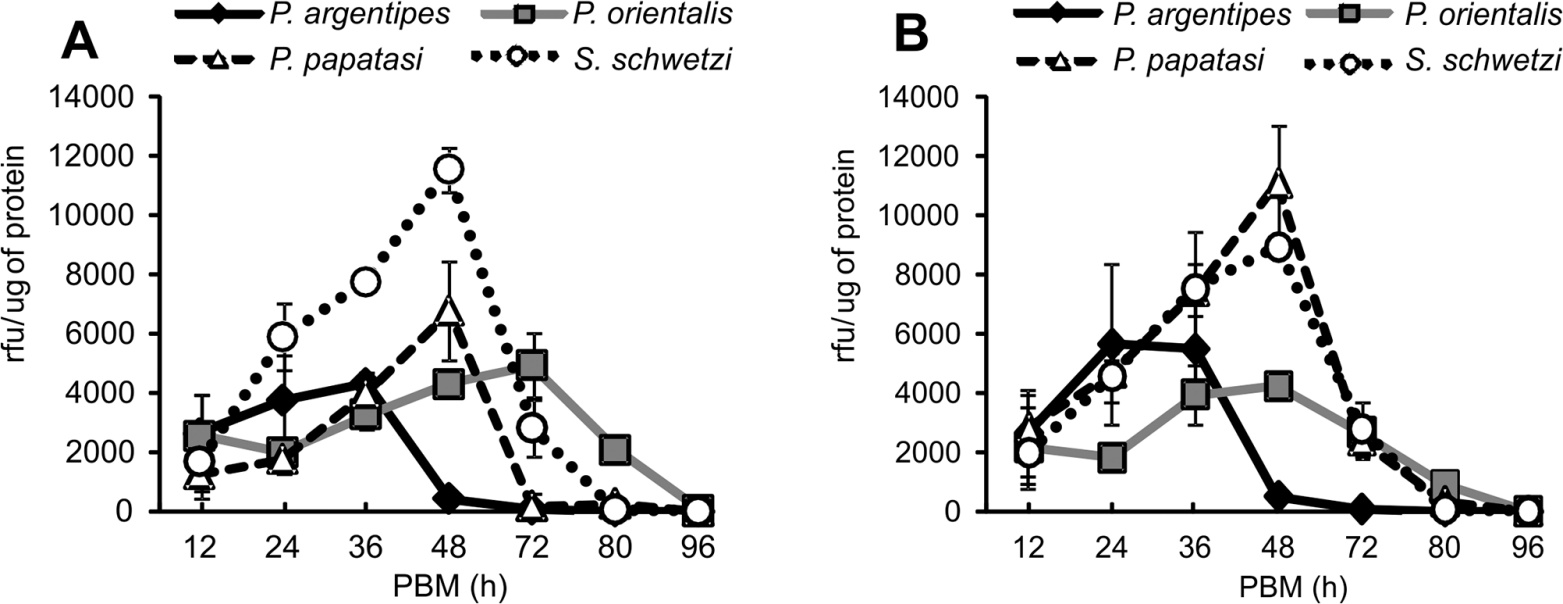
Trypsin activity in *P*. *argentipes*, *P*. *orientalis*, *P*. *papatasi* and *S*. *schwetzi* midguts. Trypsin activity was measured at 12, 24, 36, 48, 72, 80, and 96 hours PBM in midgut homogenates (c = 0.005 gut/ml) of bloodfed females using fluorogenic substrate Boc-Leu-Gly-Arg-AMC (40 μM). Data from two independent experiments were pooled. **6A:** Females fed on anesthetized BALB/c mice. **6B:** Females fed through a chick-skin membrane on heat-inactivated rabbit blood.

**Fig 7:**
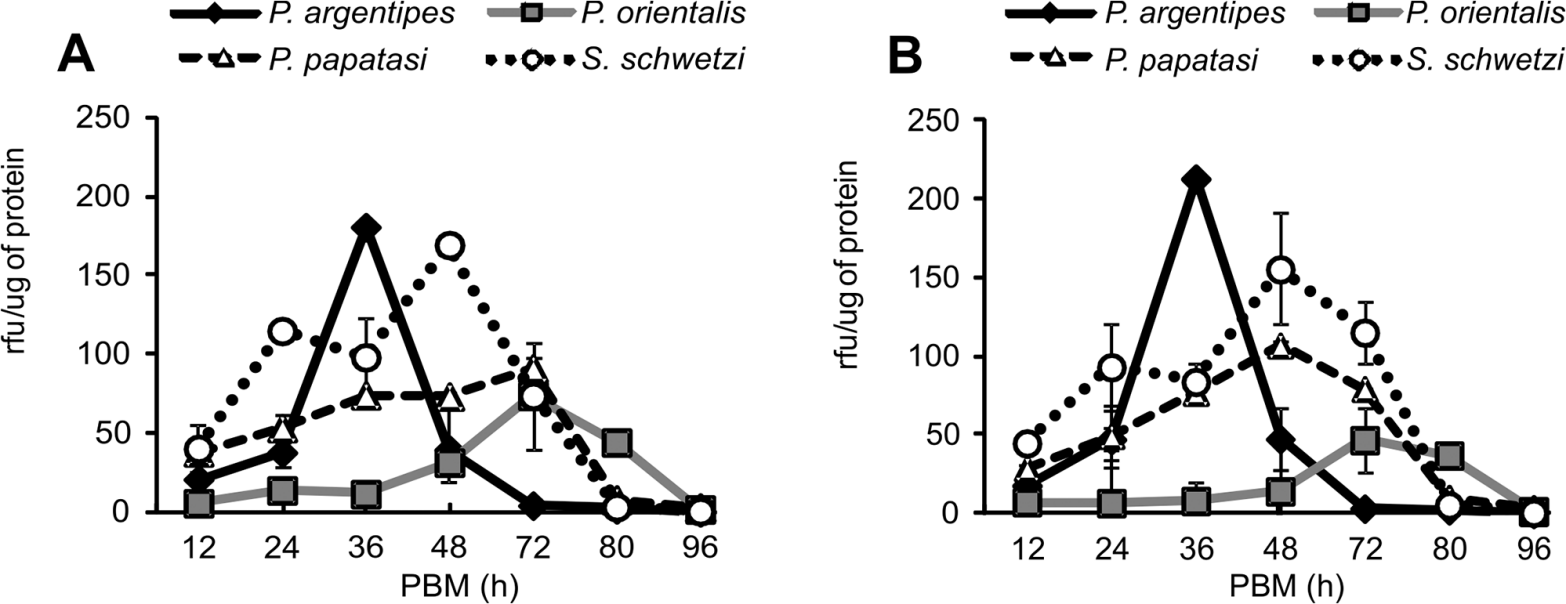
Chymotrypsin activity in *P*. *argentipes*, *P*. *orientalis*, *P*. *papatasi* and *S*. *schwetzi* midguts. Chymotrypsin activity was measured at 12, 24, 36, 48, 72, 80 and 96 hours PBM in midgut homogenates (c = 0.5 gut/ml) of bloodfed females using fluorogenic substrate Suc-Ala-Ala-Pro-Phe-AMC (40 μM). Data from two independent experiments were pooled. **7A:** Females fed on anesthetized BALB/c mice. **7B:** Females fed through a chick-skin membrane on heat-inactivated rabbit blood.

*Phlebotomus argentipes* and *P*. *orientalis* highly differed in the time course of trypsin and chymotrypsin activities. *Phlebotomus argentipes* females digested faster, with values of both enzymatic activities peaking about 24 − 36 hours PBM, and dropping to almost zero by 72 hours PBM. On the contrary, the highest values of proteolytic activities in *P*. *orientalis* females were observed between 48 and 72 hours PBM (Figs 6 and 7). In both *L*. *donovani* vectors, trypsin activity was significantly lower than in *S*. *schwetzi*. Maximum chymotrypsin activity in *P*. *orientalis* was very low compared to the other three species. In contrast, a very high peak of chymotrypsin activity was found in *P*. *argentipes* 36 hours PBM (Fig 7). In all sand fly species, the time courses of trypsin and chymotrypsin activities were analogous in females fed on mice and on rabbit blood through the chick-skin membrane (Figs 6 and 7).

### The effect of initial infective dose on *L*. *donovani* development in *P*. *argentipes*

**Fig 8:**
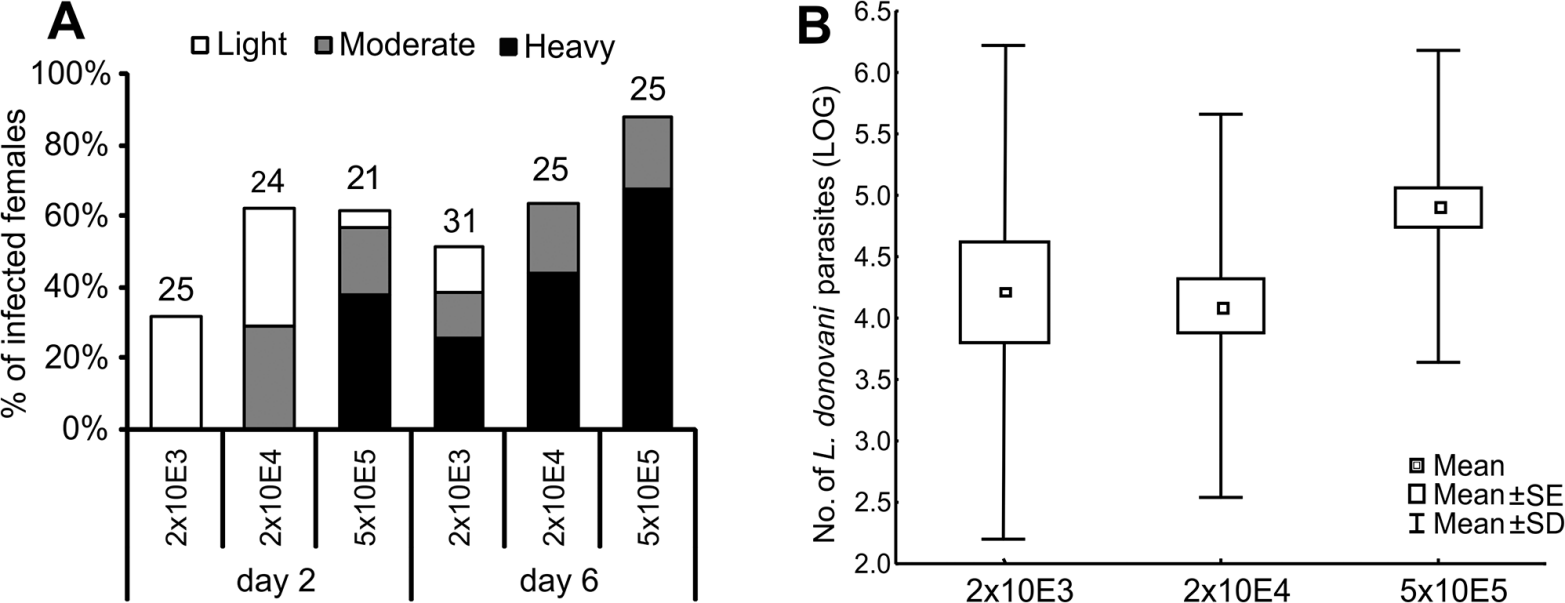
The effect of initial infective dose on the development of *L*. *donovani* in *P*. *argentipes*. Females of *P*. *argentipes* were infected by feeding on a suspension of 2×10^3^, 2×10^4^ or 5×10^5^promastigotes/ml of blood and kept at 26°C. **8A**: Females were examined microscopically 2 and 6 days post infection. Intensities of infection were classified into three categories according to their intensity: light (< 100 parasites/gut), moderate (100 − 1000 parasites/gut), or heavy (> 1000 parasites/gut). Numbers above the bars indicate the number of dissected females. **8B:** The number of parasites was measured individually by Q-PCR in 20 females of each group on day 8 PBM.

The effect of three initial infective doses on infection rates and total parasite numbers was tested in *P*. *argentipes* infected by *L*. *donovani* (strain GR374). Different parasite numbers taken by females affected their subsequent infection rates. In groups with lower initial infective doses (2×10^3^ and 2×10^4^ promastigotes/ml), the rate of late-stage infections was about 50 and 65% females, respectively, while in the group infected with 5×10^5^ promastigotes/ml the infection rate reached 90% (Fig 8A). Different initial infective doses also influenced total parasite numbers in the sand fly midgut. The Q-PCR showed significant differences (KW-H_(1;35)_ = 8.3; P < 0.01) in parasite loads at late-stage infections (day 8 PI) between the groups of females with low (2×10^3^ and 2×10^4^ promastigotes/ml) and high (5×10^5^ promastigotes/ml) infective doses. Nevertheless, the majority of positive females infected with low infective doses posed very heavy infections with more than 10 000 parasites/gut (Fig 8B).

### Comparison of the susceptibility of *P*. *orientalis* and *P*. *argentipes* to *L*. *donovani* infection

**Fig 9:**
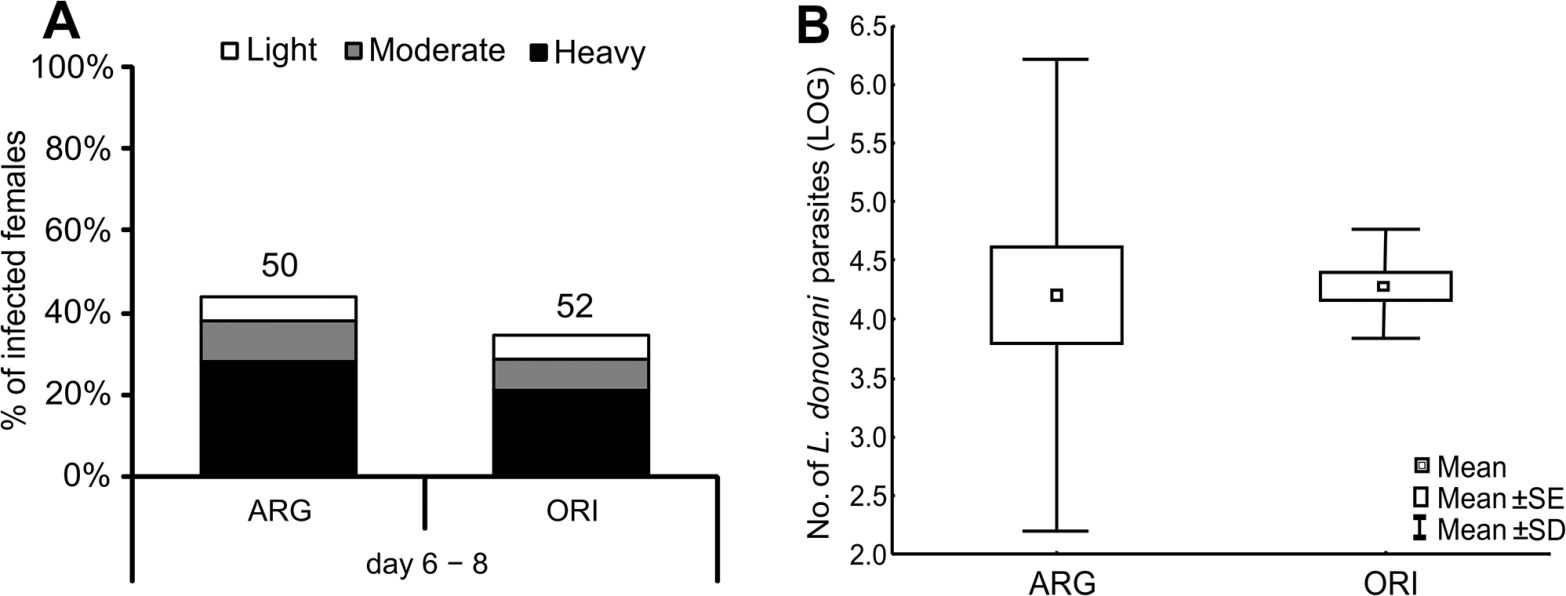
Comparison of the susceptibility of *P*. *argentipes* and *P*. *orientalis* to *L*. *donovani* Females of *P*. *argentipes* (ARG) and *P*. *orientalis* (ORI) were infected by feeding on a suspension of 2×10^3^ promastigotes/ml of blood and kept at 26°C. **9A**: Females were examined microscopically on days 6 and 8 PBM. Intensities of infection were classified into three categories according to their intensity: light (< 100 parasites/gut), moderate (100 − 1000 parasites/gut), or heavy (> 1000 parasites/gut). Numbers above the bars indicate the number of dissected females. **9B:** The number of parasites from 20 females of each group was measured individually by Q-PCR 8 days post infection.

Development of *L*. *donovani* infections initiated with 2×10^3^ promastigotes/ml was compared in *P*. *argentipes* and *P*. *orientalis*. Parasites developed similarly in both groups of females: at the late stage of infection (day 6 − 8 PI) infection rates were about 44 and 35% in *P*. *argentipes* and *P*. *orientalis*, respectively, and a majority of infected females had high parasite loads (Fig 9A). Similarly, Q-PCR revealed no significant differences (KW-H_(1;10)_ = 0.05; P = 0.83) in total parasite numbers in sand fly midguts on day 8 PI (Fig 9B).

The location of parasites during the late-stage infection was also similar in both groups of infected females, and colonization of the thoracic midgut and stomodeal valve was observed since day 6 PI. On day 8 PI, the stomodeal valve was colonized in about 80 and 70% of infected *P*. *argentipes* and *P*. *orientalis* females, respectively.

## Discussion

Experiments on two *L*. *donovani* vectors showed that *P*. *argentipes* and *P*. *orientalis* are equally susceptible to *L*. *donovani* parasites; both species infected with the very low dose (2×10^3^ promastigotes/ml) developed fully comparable infections. Light microscopy and Q-PCR did not reveal any significant differences in the development of *L*. *donovani* between these two vectors.

These results are supported by experiments focused on the effect of initial infective *L*. *donovani* doses on total parasite numbers in late-stage infections. Our previous study [30] revealed that infection rates of *P*. *orientalis* females infected with 2×10^3^ promastigotes/ml were 45%, while rates in three groups infected with 2×10^4^, 1×10^5^ or 5×10^5^ promastigotes/ml reached 75–95%; however, mature infections and colonization of the stomodeal valve were observed in all groups, including the one with the lowest dose [30]. In the present study, *L*. *donovani* promastigotes developed similarly in *P*. *argentipes*. Infection rates for groups of *P*. *argentipes* females with initial infected dose of 2×10^3^, 2×10^4^ and 5×10^5^ promastigotes/ml infections were about 50, 65 and 90%, respectively, and even the lowest initial dose resulted in very heavy late-stage infections.

Taking into account the average bloodmeal volume taken by *P*. *argentipes* and *P*. *orientalis* (0.63 μl and 0.59 μl, respectively), this initial dose corresponds to only one or two promastigotes ingested by females during bloodfeeding. This means that in both *L*. *donovani* vectors, even 1 or 2 parasites represent a sufficient dose for the initiation of mature infections with colonization of the stomodeal valve in about one half of females.

Despite the same susceptibility to *L*. *donovani*, the two natural vectors of this parasite showed several striking differences in bloodmeal digestion and secretion of the PM and digestive enzymes. Rapid defecation of bloodmeal remnants was observed in *P*. *argentipes*, where all females were defecated by day three PBM, while females of *P*. *orientalis* defecated until the fourth to sixth day PBM. This means that *L*. *donovani* promastigotes in *P*. *argentipes* had a shorter time to develop forms capable of attaching to the midgut epithelium. Wilson *et al*. [33] showed that in *Leishmania*, including the *L*. *donovani* complex, binding to the sand fly midgut is strictly stage-dependent and is a property of those forms found in the middle phase of development, long nectomonads and short nectomonads (leptomonads), but is absent in the earlier procyclic promastigote stage. This suggests that in *P*. *argentipes*, procyclic *Leishmania* parasites must transform to nectomonads much faster in than in *P*. *orientalis*.

The two principal *L*. *donovani* vectors also differ in the time course of trypsin and chymotrypsin activities. The proteolytic activities in *P*. *argentipes* peaked around 24 − 36 hours PBM, while in midguts of *P*. *orientalis* the highest proteolytic activities were found between 48 and 72 hours PBM. Peaks of trypsin activity in both refractory species *P*. *papatasi* and *S*. *schwetzi* were higher than in either of the *L*. *donovani* vectors. This finding supports previous hypotheses about trypsin as a molecule affecting *Leishmania* development in sand flies [7–9,13,14,34]. On the other hand, the peak of chymotrypsin activity in *P*. *argentipes* was much higher than in *P*. *orientalis*, which suggests that these differences in chymotrypsin activity do not play such a significant role in the vector competence of sand flies to *L*. *donovani*.

The peritrophic matrix plays several important roles during bloodmeal digestion and may also act as a barrier preventing the release of *Leishmania* from the bloodmeal to the ectoperitrophic space (reviewed by [4,20]) where they attach to the midgut epithelium during defecation [21,22,33]. *Leishmania* parasites are not able to traverse the PM before its rough disintegration which is indicated by escape of bloodmeal remnants into the ectoperitrophic space.[19] Although there were significant differences in the PM kinetics among the four sand fly species tested, the PM was present in all sand fly species during the peak of proteolytic activities and its degradation started several hours later. Interestingly, both proven vectors of *L*. *donovani* strikingly differed in the formation and degradation of the PM. In *P*. *argentipes* the PM was present only for a short time, firstly forming at 12 hours PBM and degrading between 24 and 48 hours PBM, while in *P*. *orientalis* the PM appeared by 6 hours PBM and degraded between 48 and 72 hours PBM.

Formation of the PM was more rapid in *P*. *papatasi* and *S*. *schwetzi* than in both *L*. *donovani* vectors. In all females the PM was present by 3 and 6 hours PBM in *S*. *schwetzi* and *P*. *papatasi*, respectively, while this process lasted 24 hours PBM in *P*. *orientalis* and *P*. *argentipes*. According to Pimenta *et al*. [11] the PM can protect parasites against the rapid diffusion of digestive enzymes during the early phase of infections. The addition of exogenous chitinase to the bloodmeal stopped formation of the PM in *P*. *papatasi*, which resulted in the suppression of *L*. *major* infections for 4 hours PBM [11]. However, our results show that natural *L*. *donovani* vectors form the PM later, i.e. several hours after this critical period (see above). Our data confirmed that speed of the PM formation is species-specific.

Sadlova *et al*. [24] concluded that the crucial parameter for the early-stages development of *Leishmania* in the midgut of *S*. *schwetzi* is the duration of the period between the degradation of the PM and defecation [24]. Using amastigotes-initiated infections, Sadlova and Volf [19] demonstrated that breakdown of the PM in *P*. *duboscqi* coincides with the transformation of *L*. *major* procyclic promastigotes to long nectomonads [19], which are able to attach to the midgut epithelium to avoid defecation with bloodmeal remnants. This transformation seems to be associated with the diffusion of signal molecules from the ectoperitrophic space to the parasite surroundings through a broken PM (reviewed by [3]). In the present study, there was a very short time period between the breakdown of the PM and defecation in *S*. *schwetzi* (or the intact PM was present until defecation). Previously, using promastigote-initiated infections, Sadlova *et al*. [24] described the delayed transformation of *L*. *donovani* promastigotes in *S*. *schwetzi* in comparison with the permissive vector *Lutzomyia longipalpis* on day 2 PBM. Parasites remained in the procyclic promastigote stage because of an intact PM and were defecated [24]. On the other hand, here we found that despite a quite early defecation, in *P*. *argentipes* there is about a 24 hour period on average between the breakdown of the PM and defecation. This “window” clearly provides enough time for *L*. *donovani* promastigotes to bind to the midgut epithelium. In *P*. *orientalis* and *P*. *papatasi* this period is longer (48 and 38 hours, respectively); however, *P*. *papatasi* is refractory to *L*. *donovani* due to the lack of a surface ligand for parasite LPG (reviewed by [9,10,23]).

## Conclusions

Differences found in most parameters of bloodmeal digestion of various sand fly species appear not relate to the known differences in vectorial competence. In spite of the fact that *P*. *argentipes* and *P*. *orientalis* are both natural vectors of *L*. *donovani*, they have completely different time courses of bloodmeal digestion. Females of *P*. *argentipes* possess fast bloodmeal digestion with a very high peak of chymotrypsin activity, rapid degradation of the PM and defecation finishing already on day three PBM, while *P*. *orientalis* females digest considerably slower, have low peaks of proteolytic activities and defecate around day five PBM. However, our results indicate that both vectors are similarly susceptible to experimental infection with *L*. *donovani* (GR374) and even one or two *Leishmania* parasites are sufficient for the establishment of mature late-stage infections in *P*. *argentipes* and *P*. *orientalis*.

## Supporting Information

S1 Table Tukey´s Multiple Comparison Test table for bloodmeal volume. (DOCX)
