# Supplementary material for: Comparison of Bloodmeal Digestion and the Peritrophic Matrix in Four Sand Fly Species Differing in Susceptibility to Leishmania donovani
Source: PLoS One. 2015 Jun 1;10(6):e0128203. doi: 10.1371/journal.pone.0128203 (PMC4452187; doi:10.1371/journal.pone.0128203)
Supplement: S1 Table — (DOCX) [file pone.0128203.s001.docx]

|  | *P. argentipes*  0.68000 | *P. orientalis*  0.55500 | *P. papatasi*  0.77500 | *S. schwetzi*  0.86500 |
| --- | --- | --- | --- | --- |
| *P. argentipes* |  | 0.130508 | 0.330836 | **0.011882** |
| *P. orientalis* | 0.130508 |  | **0.002491** | **0.000190** |
| *P. papatasi* | 0.330836 | **0.002491** |  | 0.377196 |
| *S. schwetzi* | **0.011882** | **0.000190** | 0.377196 |  |
